# Structural variation of centromeric endogenous retroviruses in human populations and their impact on cutaneous T-cell lymphoma, Sézary syndrome, and HIV infection

**DOI:** 10.1186/s12920-019-0505-8

**Published:** 2019-05-02

**Authors:** Mark H. Kaplan, Mark Kaminski, Judith M. Estes, Scott D. Gitlin, Joseph Zahn, James T. Elder, Trilokraj Tejasvi, Elizabeth Gensterblum, Amr H. Sawalha, Joseph Patrick McGowan, Michael H. Dosik, Haner Direskeneli, Guher Saruhan Direskeneli, Sally N. Adebamowo, Clement A. Adebamowo, Mohammad Sajadi, Rafael Contreras-Galindo

**Affiliations:** 1 Division of Infectious Diseases, Department of Internal Medicine, University of Michigan, Ann Arbor, MI 48109 USA; 2 Division of Hematology/Oncology, Department of Internal Medicine, University of Michigan, Ann Arbor, MI 48109 USA; 3 Division of Dermatology, Department of Internal Medicine, University of Michigan, Ann Arbor, MI 48109 USA; 4 Division of Rheumatology, Department of Internal Medicine, University of Michigan, Ann Arbor, MI 48109 USA; 5 Division of Infectious Diseases, The Feinstein Institute for Medical research, Manhasset, NY 11030 USA; 6 Hematology Oncology Associates, Setauket, NY 11776 USA; 7 Division of Rheumatology, School of Medicine, Marmara University, Istanbul, Turkey; 8 Department of Physiology, Istanbul Faculty of Medicine, Istanbul University, Istanbul, Turkey; 9 Department of Epidemiology and Public Health, University of Maryland School of Medicine, Baltimore, MD 21201 USA; 10 Greenebaum Comprehensive Cancer Center, University of Maryland School of Medicine, Baltimore, MD 21201 USA; 11 Institute of Human Virology, University of Maryland School of Medicine, Baltimore, MD 21201 USA; 12 Ann Arbor Veterans Affairs Hospital, Ann Arbor, MI 48105 USA; 13 Hormel Institute, University of Minnesota, Austin, MN 55912 USA

**Keywords:** HERV-K, K111, Pericentromeric instability, Centromeres, CTCL, AIDS, HIV

## Abstract

**Background:**

Human Endogenous Retroviruses type K HML-2 (HK2) are integrated into 117 or more areas of human chromosomal arms while two newly discovered HK2 proviruses, K111 and K222, spread extensively in pericentromeric regions, are the first retroviruses discovered in these areas of our genome.

**Methods:**

We use PCR and sequencing analysis to characterize pericentromeric K111 proviruses in DNA from individuals of diverse ethnicities and patients with different diseases.

**Results:**

We found that the 5′ LTR-*gag* region of K111 proviruses is missing in certain individuals, creating *pericentromeric instability*. K111 deletion (−/− K111) is seen in about 15% of Caucasian, Asian, and Middle Eastern populations; it is missing in 2.36% of African individuals, suggesting that the −/− K111 genotype originated out of Africa. As we identified the −/−K111 genotype in Cutaneous T-cell lymphoma (CTCL) cell lines, we studied whether the −/−K111 genotype is associated with CTCL. We found a significant increase in the frequency of detection of the −/−K111 genotype in Caucasian patients with severe CTCL and/or Sézary syndrome (*n* = 35, 37.14%), compared to healthy controls (*n* = 160, 15.6%) [*p* = 0.011]. The −/−K111 genotype was also found to vary in HIV-1 infection. Although Caucasian healthy individuals have a similar frequency of detection of the −/− K111 genotype, Caucasian HIV Long-Term Non-Progressors (LTNPs) and/or elite controllers, have significantly higher detection of the −/−K111 genotype (30.55%; *n* = 36) than patients who rapidly progress to AIDS (8.5%; *n* = 47) [*p* = 0.0097].

**Conclusion:**

Our data indicate that *pericentromeric instability* is associated with more severe CTCL and/or Sézary syndrome in Caucasians, and appears to allow T-cells to survive lysis by HIV infection. These findings also provide new understanding of human evolution, as the −/−K111 genotype appears to have arisen out of Africa and is distributed unevenly throughout the world, possibly affecting the severity of HIV in different geographic areas.

**Electronic supplementary material:**

The online version of this article (10.1186/s12920-019-0505-8) contains supplementary material, which is available to authorized users.

## Background

The Human Endogenous Retrovirus HERV-K HML-2 (HK2), first discovered in teratocarcinoma cell lines [1] and sequenced in 1986 [2], is one of the most recent retrovirus groups to have entered the genome of the primate lineage. They arose through a number of single exogenous infections of germ line cells over the last 35 million years of primate evolution [3–14]. HK2 proviruses, which use a Lys tRNA as a primer for reverse transcription, were discovered in human cells due to their similarity to the Mouse Mammary Tumor Virus (MMTV). These proviruses have 5′ and 3′ long terminal repeats (LTRs) that were identical at the time of integration but accumulated mutations over time. Some HK2 entered the genome after the Homo-Pan divergence about 6–8 million years ago, and therefore are only found in either humans or chimpanzees [13]. With time, most of these integrated proviruses became silenced by introduction of stop codons, frame shifts, and indels mutations. One major type of inactivation originated by recombination between the 5′ and 3′ LTRs of full-length proviruses, creating hundreds of solo LTRs. Utilizing the human genome assembly (GRCh37/hg19), early estimates suggested that there are approximately 91 full-length HK2 proviruses and 944 solo LTRs [15]. At least 11 of these HK2 are polymorphically inserted into the human genome [5, 10, 12, 13, 15–18]. Mining of data generated by Next Generation sequencing from the Cancer Genome Atlas Project and the WGS500 project uncovered 17 new HK2 proviruses. Some of these HK2 proviruses are present polymorphically in only 2 of 358 individuals studied and some in over 95% of individuals [19]. More recent mining of data from the 1000 Genome project and the Human Genome Diversity Project [20] revealed the existence of 36 more HK2 proviruses, which were found to be present in < 0.05 to 75% of the different populations studied. Five of these sites represented new solo LTRs. One new HK2 provirus, Xq21.33, has intact ORFs for all viral genes and is polymorphically present in Pygmy and Nigerian populations [20]. Only two other nearly intact proviruses, notably K113 [21, 22] and K115 [23] theoretically could also be able to produce infectious viral particles, but studies have shown these viruses are not capable of replication.

Recently, we discovered three new type 1 HK2 endogenous retroviruses, which we named K111, K222, and a recombinant virus K111/K222 [24–26]. Unlike the other 117 known proviruses, these newly discovered proviruses are found in the centromeric and pericentromeric regions of the human genome, restricted to 15 specific chromosomes. They are somewhat similar to K105 [5] and K112 sequences previously reported [27]. K111 proviruses are mostly present in the centromeric region of chromosomes 21 and 22 [25]. The progenitor of K111 appears to have integrated before the Homo-Pan divergence and expanded in copy number during the evolution of hominids but not the chimpanzee. K111 is present in the chimpanzee as one copy in the telomere of chromosome 7, but it is found in about 5 copies in the Denisovan and Neanderthal genomes [28–31] and in several hundred copies in modern humans [25]. Shortly after we discovered K111, we found another centromeric endogenous provirus that we named K222, which is present mostly in the pericentromeric region of chromosomes 13, 14, and 15 [26]. K222 infected the germ line approximately 25 million years ago and is present as a single copy in the genomes of high order primates [26]. K111 and K222 share some homologous regions, and some recombinant sequences of these two proviruses exist in the centromeres as well. It is not yet known how many K111, K222, and recombinant K111/K222 proviruses actually exist, but based on quantitative assays and deep sequencing we have estimated that there may be up to several hundreds to thousands of such proviruses present in the germ line of modern man [25].

In contrast to the previously known 117 proviruses, which spread mostly by infection, K111 and K222 proviruses appear to have spread in human centromeres via homologous recombination [24–26]. K222 lacks the 5′ LTR and *gag* gene, and is inserted into a pericentromeric repeat, termed pCER [26]. K111 proviruses have a 5′ and 3′ LTR and mostly inactive *gag*, *pol* and *env* genes. K111, which is a Type 1 HK2, however, has an intact ORF for the accessory gene *np9* [32–36]. This oncogene arose after the split of Hominids from old world primates and is present in gibbons [33], gorillas, orangutans, chimpanzees and humans. It makes a 74 amino acid protein called Np9, which binds to the ubiquitin ligase MDM2 and inhibits its activity towards p53 [33]. It also acts as a molecular switch for co-activating beta-catenin, ERK, Akt and Notch1, and promotes the growth of human leukemia stem/progenitor cells [34]. Np9 also interacts with the promyelocytic leukemia zinc finger protein (PLZF), a tumor suppressor and transcriptional repressor [35], as well as with Ligand of numb protein X (LNX), ultimately increasing the transactivation activity of Notch and likely affecting tumorigenesis [36]. Interestingly, there are different forms of *np9* transcripts produced by K111 s that come from their different sequence variants [32]. While the function of the canonical Np9 has begun to be elucidated, nothing is known about the function of Np9 s expressed by K111.

In our original studies of K111, we found that a patient with Sézary syndrome (Sz), a severe form of cutaneous T-cell lymphoma (CTCL), was missing K111 proviruses from her genome. We also discovered that the CTCL cell line Hut 78, and a derivative of that cell line, H9 [37], that was developed to grow HIV at high concentrations, was also missing K111, a genotype we refer as −/−K111 in this study. A −/− K111 genotype would also indicate that hundreds or thousands of K111 proviruses are missing from the pericentromeres creating *pericentromeric instability*, a concept defining the high frequency of deletions in the pericentromeric area of our genome. As a result, we decided to study whether patients with CTCL have a −/−K111 genotype. In addition, we studied the prevalence of the −/−K111 genotype in human populations and the association of the −/−K111 genotype with disease entities of humans. We also further characterized K111 sequences in chromosomes 21 and 22 to better define the heterogeneity of K111 s in human centromeres.

## Methods

### Study samples

DNA samples from Human/Rodent somatic hybrid cell lines (each one containing a single human chromosome) and their parental rodent cells were obtained from the NIGMS Human/Rodent Somatic Cell Hybrid Mini Mapping Panel # 2 DNA, Coriell Cell Repositories.

DNA samples from people of different genders and ethnic origins were obtained from the HRC2 Human Random Control DNA panel 2 (Sigma Aldrich, 96 Caucasian people) and from Human variation panels HD03 (Indo Pakistani), HD05 (Middle Eastern), HD07 (Japanese), HD12 (Africans South of the Sahara), HD20 (Russian Krasnodar), HD21 (Italian), HD22 (Ashkenazi Jewish), HD32 (Chinese), and samples from Mb pygmy (NA10492, NA10493, NA10494, NA10495, NA10496) from the Coriell Cell Repositories; these DNA samples were previously used in another study [26].

DNA from a cohort of HIV patients was obtained from the peripheral blood lymphocytes (PBLs) of patients cared for at the University of Maryland, Baltimore, MD; North Shore University Hospital, Manhasset, NY; and the University of Michigan, Ann Arbor. Other samples from HIV-infected individuals were obtained from the PBLs and or EBV transformed cells from patients enrolled in the MACS, a natural history study of men who reported having sex with men. The study participants were over age 24 to age 79.

Samples of whole blood and/or PBLs extracted from whole blood were also obtained from patients with breast cancer, different lymphomas, cutaneous T-cell lymphoma (CTCL), chronic lymphocytic leukemia (CLL), and normal controls at the University of Michigan Health System and the VA Ann Arbor Healthcare System, Ann Arbor, MI, and/or at North Shore Hematology/Oncology Associates, East Setauket, NY. All patients had pathologically confirmed cancers. The patients in the study were over age 30 to age 82.

DNA from either PBLs and/or PBLs transformed by EBV from Caucasian patients with known psoriasis was obtained from a cohort of patients cared for at the University of Michigan Dermatology Department, ages 22 to 73 years. DNA extracted from peripheral blood mononuclear cells (PBMCs) from selected patients with known lupus erythematosus and with Takayasu arteritis was obtained from patients in studies at the Division of Rheumatology, University of Michigan, and Mamara and Istanbul University in Istanbul, Turkey, ages over 30 years. DNA from PBLs of patients from Nigeria, ages 35 to 72 years, was obtained from a study of patients with breast cancer in Nigeria conducted at the Institute of Human Virology, University of Maryland, Baltimore.

All patients with CTCL had biopsy-proven disease, with biopsies reviewed at the University of Michigan. Two patients with Sézary syndrome (Sz) were cared for elsewhere, one at the University of Michigan and the Dana Farber Hospital, Boston, MA and a second one (our index case) at Yale University, New Haven, CT and North Shore University Hospital Manhasset, NY. Patients considered to have Sz had evidence of circulating atypical T-cells (Sézary cells) early in disease, which were usually "CD4+, CD45RO+ with frequent loss of T-cell surface antigens CD2, CD5, and/or CD7 [38]. Most circulating Sézary cells are CD4+, CD7-, and CD26-. There was usually a CD4+/CD8+ ratio of > 6, often with greater than 1000 aberrant cells present in circulation. Epidermotropism, Pautrier’s abscess, and haloed lymphocytes were not common. Classic cutaneous T-cell lymphoma (CTCL) was defined pathologically as consisting of a proliferation of mature CD4 + CD45RO+ memory T-cells early in disease. In almost all cases, there was a confirmatory diagnostic test for T-cell clonality with alpha/beta or gamma/delta T-cell receptor (TCR) gene rearrangements [39–43]. Large cell transformation (LCT) was determined when patients with pre-existing CTCL showed evidence of a morphologic change in small to medium sized atypical T-cell lymphocytes to a large cell variant, usually in > 25% of the lymphocyte population. These cells are typically CD30+, but can be also CD30- with usually no more than 75% of these cells having large cell morphology. These cells often show folliculotropism [44, 45].

We obtained samples from HIV-infected patients with different rates of disease progression in the MACS cohort and from other institutions. For the purposes of this study, we will use the term LTNPs to include: 1. *Elite Controllers* (*EC*), who had a viral load of less than 50 HIV RNA copies/ml on 2 or more occasions within 1.5 years in the absence of therapy (one detectable measurement of less than 1000 copies/ml was allowed between the two suppressed values). 2. *Viremic Controllers* (*VC*), who had a viral load of less than 2000 HIV RNA copies/ml on two or more tests within 1.5 years in the absence of therapy (no viral load spikes were allowed). 3. Other *Long-Term Non-Progressors (LTNPs),* patients who remained AIDS-free without therapy for 15 or more years. A second category of patients are called Intermediate Progressors*, and* are people who developed AIDS 5 to 12 years following seroconversion. The third category, Rapid Progressors*,* defines patients who developed AIDS within 3 years of seroconversion in the MACs cohort or from transfusion 3 years earlier at our combined institutions. It also includes patients we call *First AIDS* who appeared with an opportunistic infection, a severe neurological disease, a lymphoma or Kaposi sarcoma, and/or a CD4 count of less than 200 cells/mL of blood when they were first seen. In some patients, treatment with anti-retroviral drugs prevented determining the natural history of the disease. These samples comprise our fourth category, which we call *“*Unclassified*”*.

### Cell lines

The following cells lines were utilized in this study: Hut 78, and derivatives of Hut 78, notably H9 and H9/HTLVIII. These cell lines were obtained from the AIDS Research and Reference Reagent Program. Cells were maintained in RPMI medium and supplemented with 10% fetal bovine serum. Human/Rodent somatic hybrid cell lines were obtained from a human chromosomal DNA mapping panel (NIGMS Human/RodentSomatic Cell Hybrid Mini Mapping Panel # 2 DNA Coriell Cell Repositories).

### DNA extraction

DNA was extracted from PBLs and/or cell lines using the DNeasy blood and tissue kit™ (Qiagen, http://www.qiagen.com) or from whole blood or cell lines using the Gentra Puregene Blood Kit™ (Qiagen).

### Detection of K111 insertions

Centromeric K111 s were amplified by PCR with the primers P1, which binds to K111 flanking centromeric repeat CER:D22Z3, and primer P4, which binds to the K111 *gag* gene to produce a ~ 1.6 Kb amplification product [25]. The PCR was carried out using the Expand Long Range dNTPack PCR kit (Roche Applied Science, Indianapolis, IN). PCR was performed at a final volume of 50 μl using an initial step of 92 °C for 2 min, followed by 35 PCR cycles consisting of denaturation at 92 °C for 15 s, annealing at 55 °C for 30 s, and extension at 68 °C for 5 min. Amplification products were confirmed by sequencing [25].

### Walking the 5′ genome of K111

PCR amplification was carried out using the primer P1 that binds to the K111 flanking centromeric repeat CER:D22Z3 and reverse primers located on the *gag*, *pro*, and *pol* of HK2, called P4, K111 986R, K111 1584R, K111 2499R, and K111 3460R. The amplification conditions are the same as those used above.

### Long-range amplification and sequencing of K111

Production of K111 long sequences of chromosome 21 and 22 for Pac Bio sequencing were carried out using the primer set K111 5359F and P2R. Long sequences for CTCL and HIV samples and cell lines HUT 78 and H9 were produced using a primer set P1 and K111 2499R that binds to the integration site of K111 and to the *gag* region of K111, or a set of primers K111 6353F and P2R that bind to the *env* gene of K111 before the splice donor site of *np9* [30] and the 3′ integration site, respectively. Amplifications were carried out with four different bar codes attached to the primer K111 2499R or to K111 6353F, respectively. Reactions were carried out in 50 μL using 250–500 ng of total DNA per reaction with the following conditions: 92 °C for 2 min, and 40 cycles consisting of 92 °C for 15 s, 55 °C for 15 s, and 68 °C for 5 min using the Expand Long Range dNTPack PCR kit (Roche Applied Science, Indianapolis, IN).

The bar-coded PCR products were size-selected and purified using the Blue Pippin gel analyzer. The chromosomal products were purified using similar conditions. Adapters were ligated onto the PCR products and the libraries were sequenced using the P6 polymerase kit and C4 sequencing reagents on a PacBio RS II SMRT DNA Sequencing System. The sequences were processed using the PacBio SMRT portal. The sequences were de-barcoded and processed using the RSReadsOfInsert software filtering for sequences that pass five times around each SMRT cell to obtain quality values ~ 99.9%. Sequences were de-duplicated and the edited sequences were aligned to the K111 reference genome (Acc No. GU476554.2) using the RS Resequencing and Quiver v1 parameters. In order to determine sequence variants of proviruses among human populations we used the minor variant calling platform in the SMRT portal for reads aligned to K111 consensus sequence that have passed five times around each SMRT cell. The sequence alignments were visualized in the IGV viewer.

### Real-time qPCR specific for centromeric K111 and K222

The copy number of K111 found in the cellular DNA was measured by qPCR using the primer K111 1584F and a modified primer K111 1701R that has locked nucleic acid (LNA) in the CAA sequence underlined in the primer sequence below, which is highly specific for K111 to produce a 117 bp product that is readily detected using Sybrgreen. This LNA primer detects specific mutations in K111 *gag* but not other HK2s [25]. The qPCR was performed using the goTaq® qPCR master mix in a reaction volume of total 20 μl. The following PCR conditions were used: 10 min at 95 °C, followed by 50 cycles consisting of 30 s of denaturation at 95 °C, and 15 s of annealing/hybridization at 60 °C. The PCR reactions were run over 2 h. The relative K111 copy number was estimated using serial dilutions of DNA from one patient with the highest K111 copy number, starting at a concentration of 1000 ng per reaction. It was felt that total human DNA from a +/+K111 CTCL patient would be a more robust standard than K111 clone DNA. This DNA was frozen in small aliquots and used once in each run after defrosting. The specific detection of K111 was verified by sequencing analysis of the PCR product. The copy number of K222 in cellular DNA was measured by qPCR using a probe that specifically discriminates the K222 pCER-*pro* boundary as previously described [25].

### Statistical analysis

Statistically significant differences in the mean copy number (K111 and K222 copies/genome calculated by qPCR in each group) were determined using the Student’s t-test. Frequencies of detection of K111 5′ LTR among the groups were compared by chi-square (X^2^) analyses. Two-tailed *p* values were considered significant at *p* < 0.05.

### List of primers

- P1F: 5′-ACA TTC AGA CCA TGG TAG CCG TGT -3′
- P2R: 5′-ACA GTG CTG TGT GGG TCT GAA TGA -3′
- P4R (K111 986R): 5′-GTA CCT TCA CCC TAG AGA AAA GCC T -3′
- GAPDHF: 5′-TGC ACC ACC AAC TGC TTA GCA CCC-3′
- GAPDHR: 5′-CTT GAT GAC ATC ATA TTT GGC AGG-3′
- K111 1584F: 5′-TCC TTA AGG TCA TAG TGG AGT TGT TGG TAT AC-3′
- K111 1701R LNA: 5′-CAT AAG CAT AGC TTT ATG CAA AC-3′
- K111 1965R: 5′-TCC AGG TGC CAT CGG TTG CAT-3′
- K111 2499R: 5′-TTG AGC AAC ATC TTG GAG CCT TGC-3′
- K111 3460R: 5′-ACT TGC CCA ATA TGC AGC CTT TCC-3′
- K111 6353F: 5′-GAC ATG GGA AAT AGG GAA GGT AAT A-3′
- K111 5359F 5′-TCG GCT CAA AGA GCA GAG ATG GTT-3′
- K111F: 5′-AAG AGC ACC AGG ATG CTT AAT GCC-3′
- K111R: 5′-AGT GAC ATC CCG CTT ACC ATG TGA-3′
- K111P: 5′-FAM-TGC CGG TCC TAA CAG TAG ACT CAC-BHQ1–3′
- K222F: 5′-CAG CGT TCT GGA ATC CTA TGT-3′
- K222R: 5′-TGT ATT GTG GTA ACT GGG TAT ATG T-3′
- K222P: 5′-FAM- ACC CAC ATG GCA GTG TTC TGG ATT-BHQ1–3′
- Bar codes used for Pac Bio Sequencing;
- **B** ACATCG, **E** CACTGT, **G** GATCTG, **J** AAGCTA

## Results

### Detection of a −/−K111 [Δ LTR-*gag*] genotype in CTCL patients

**Fig. 1:**
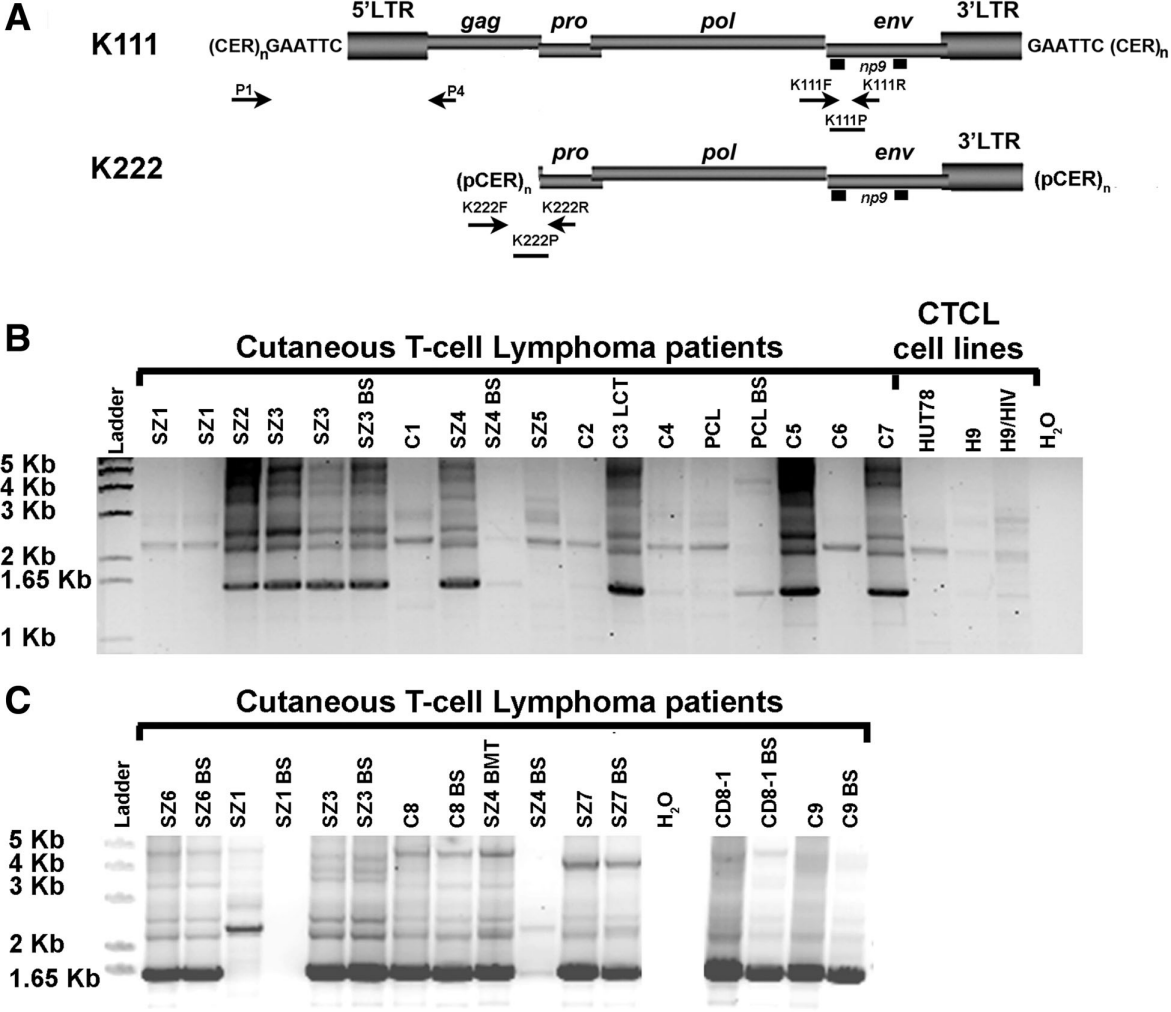
Detection of centromeric K111 in the DNA of CTCL patients and cell lines. **a** Genomic organization of K111 and K222 and primers used for PCR are shown with arrows. P1 in the alpha repeat CER: D22Z3 and P4 in K111 *gag* detect the “+/+ K111 genotype”. K222F is in the flanking pCER element and K222R binds the *pro* portion of the K222 provirus. K222P binds to the boundary of the integration site and *pro* (**b**) K111 DNA from PBLs and buccal swabs (BS) from patients with either CTCL or Sézary syndrome or CTCL with large cell transformation labeled or CTCL that transformed to peripheral T-cell lymphoma labeled C, Sz, C3LCT or PCL respectively was amplified with primers P1 and P4. A strong band shown by an arrow at mw 1614 bp represents the +/+K111 genotype. Patients Sz1, C1, Sz5, C2, C4, PCL, and C6, as well as the cell line DNA from HUT78, H9 and H9/HTLVIII, have the −/− K111 genotype and show a 2279 bp product of HERV-K (HML-2) 3p25.3 amplified non-specifically with the primers P1 and P4 when K111 is not present. Patient SZ4 shows a faint 2279 bp band in her BS while her PBLs have the characteristic 1614 bp band. Bands of higher molecular weight represent other insertions of K111 into other centromeric repeats [25]. **c** The DNA from the PBL of selected patients is compared to that from a buccal swab (BS). Most patients show the same K111 amplification pattern in their PBLs as in their BS, indicating that the K111 is in the germ line. Patient SZ4 shows a −/−K111 genotype in her BS, while her PBLs derived from her bone marrow donor has a +/+K111 genotype. Patient CD8–1 is a patient with CD8 CTCL who is not part of this study, but illustrates the pattern produced from PCR of the DNA of other non CTCL patients have using P1 and P4 primers

As we discovered the −/−K111 genotype in CTCL cells, we collected DNA from patients with CTCL and Sz (an aggressive form of CTCL) to determine whether the lack of centromeric 5′ K111 LTR-*gag* (−/−K111 genotype) is prevalent in this disease. DNA from CTCL patients was screened for the K111 provirus using a forward primer P1, which binds to the flanking CER:D22Z3 region, and a reverse primer P4, which binds in K111 *gag* (Fig. 1a). This set of primers amplifies centromeric K111 proviruses in 15 human chromosomes [24, 25]. The PCR reaction amplifies multiple products, but the major band, which we call the K111 *beta* band, at a molecular weight of ~ 1614 bp (Fig. 1b), represents multiple centromeric K111 proviral sequences from many chromosomes as determined by cloning and sequencing of this amplification product in our previous study [25]. Several CTCL patients did not show the characteristic broad K111 *beta* band (Fig. 1b and c). When the DNA samples lack the K111 *beta* band, a non-specific amplification product of ~ 2279 bp is detected, which corresponds to the LTR of HK2 3p25.3 inserted in chromosome 3, as determined by sequencing (Fig. 1b and c). Patients with a −/−K111 genotype also do not have the characteristic higher molecular weight gamma and delta bands, which are other K111 proviral sequences flanked by longer centromeric CER elements [25]. A −/− K111 genotype is also seen in the Hut 78 cell line and derivatives of Hut 78, notably H9 and H9/HTLVIII (Fig. 1b). We previously showed that using 4 additional primers that bind to other areas of the 5′ flanking region of K111 still did not produce amplification products in −/− K111 cells. This confirmed that the −/− K111 genotype observed with the primers P1 and P4 is not the result of mutations in the 5′ flanking area preventing the binding of the P1 primer during PCR, but is due to absence of K111 provirus [26].

The patients with CTCL who were in this study were characteristically much sicker than ordinary CTCL patients having failed UV light treatments and/or topical steroids. They ultimately required more invasive therapies (Additional file 1: Table S1 and Additional file 2: Table S2) to control refractory skin tumors, or they had developed large cell transformation (LCT), new cancers, or uncontrolled Sz.

Interestingly, the frequency of detection of the −/−K111 genotype in these patients was significantly higher (*p* = 0.0083) in individuals with CTCL (37.14%; *n* = 35) than in those of healthy individuals of the same ethnicity (15.6%; *n* = 160) (Table 1), suggesting that a −/−K111 genotype is associated with severe CTCL. The frequency of detection of the −/−K111 genotype was significantly higher in patients with the progressive stages of the disease such as Sz (45.5%) [*p* = .039; *n* = 11], in contrast to those that have uncomplicated but still severe CTCL (30.7%).

**Table 1:** Frequency of Detection of K111 in DNA from Cutaneous T-cell Lymphoma (CTCL) patients

Disease	No. tested	−/− K111	Frequency of −/− K111 (%)	*X* ^*2*^	*p* value
CTCL Total	35	13	37.14	6.96	0.0083
CTCL	13	4^b^	30.7	0.32	ns
CTCL + LCT	11	4^c^	36.4	2.97	0.0423
Sézary Syndrome	11^a^	5	45.5	4.260	0.0390
Caucasian CTCL	35	13	37.1	8.475	0.003601
African American CTCL	4	0	0	24	ns
Caucasian healthy	160	25	15.6		

The table shows the frequency of detection of the −/−K111 genotype in patients with CTCL. This proportion is compared to the prevalence of −/−K111 in a population of healthy Caucasian individuals. ^a^Two patients with Sz also developed LCT. Neither of these patients was −/− K111. ^b^One patient developed peripheral T-cell lymphoma and a second patient developed Hodgkin Lymphoma.^c^One patient developed concomitant Hodgkin Lymphoma. *LCT* Large cell transformation, *Sz* Sézary Syndrome. *p* values were calculated using a *X*^2^ test

Some CTCL patients also developed multiple other cancers, as shown in Additional file 1: Table S1 Patients who have the −/−K111 genotype had more secondary cancers (11/13, [84%]) in contrast to those who are +/+ K111, (17/26 [62.9%]), but this was not statistically significant (X^2^: 1.58, *p* = 0.20; Additional file 1: Table S1). Patients who are −/−K111 were less likely to have formed tumor nodules and/or large plaques that require multiple sessions of radiation therapy (4/13 [30.8%] receiving 18 treatments) than those with a +/+K111 phenotype (15/26 [52.7%] receiving as many as 59 treatments), but this was not statistically significant (X^2^: 2.51, *p* = 0.1128). During each treatment period of radiation, patients had from 1 to > 5 areas of skin radiated. On the other hand, the mortality of −/−K111 patients was higher (61.5% *n* = 8) than +/+K111 (34.6% *n* = 9) and these patients needed whole body radiation (4/13 30.7%) vs. (5/26 19.2%) as well as bone marrow transplantation (2/13 15.3% vs. 3/26 11.5%) to control disease, again all not statistically different.

Having a −/−K111 is uncommon, and indicates that K111 proviruses are completely absent from the centromeric regions of these CTCL patients, something we also refer to in this paper as *pericentromeric instability*. As we do not know whether or not the K111 genotype might vary among the cell types of the body, we obtained buccal mucosal swabs (BS) to search for the presence of K111 in healthy cells from some CTCL patients. As seen in Fig. 1c, the pattern of detection of the K111 genotype was similar in the buccal mucosal and the peripheral blood lymphocytes (PBLs) DNA, indicating that the lack of K111 or *pericentromeric instability* is found in the germ line of these patients. Of interest, one patient who had severe Sz, and who received an allogeneic bone marrow transplant, had a +/+K111 genotype detected in her transplanted peripheral blood lymphocytes (PBLs) but had a −/−K111 genotype in her buccal mucosal cells (Patient SZ4, Fig. 1b and c). We could deduce then that these +/+K111 PBLs represent the engrafted cells from her transplant.

### Detection of −/−K111 genotype in diverse populations and medical diseases

To put the loss of K111 s or *pericentromeric instability* in CTCL patients in perspective, we were interested in knowing the prevalence of the −/−K111 genotype in diverse populations. To do so, we obtained a panel of DNA from healthy Caucasian individuals from England [26] and DNA samples from people of different ethnicities around the world (Table 2). We also collected PBLs and isolated DNA from healthy Caucasian persons as well as from African patients from Nigeria and from Hispanic individuals as shown in Table 2.

**Table 2:** Frequency of Detection of K111 in DNA from individuals of different ethnic groups

Ethnicity	No. tested	−/− K111	Frequency of −/− K111 (%)	*X* ^*2*^	*p value*
Caucasian	180	29	16.11		
English	96	13	13.54		
American	64	12	18.75		
Italian	10	3	30		
Ashkenazi	10	1	10		
African total	127	3	2.36	14.1415	0.000169*
South Sahara	9	0	0	1.65	ns
Pygmy	5	0	0	0.921	ns
Nigerian	113	3	2.65	12.1039	0.000503*
Asian total	54	12	22.22	1.2288	ns
Asian (MACS)	25	6	24	1.0872	ns
Chinese	10	2	20	0.0506	ns
Japanese	10	1	10	0.3408	ns
Southeast Russia	9	3	33.3	1.933	ns
Amerindian (MACS)	21	3	14.3	0.0255	ns
African American	39	3	7.6	1.82	ns

Stars indicate significantly decreased detection of the −/− K111 genotype in Africans, including Nigerian individuals, as compared with the Caucasian population. *p* values were calculated using the *X*^2^ test

We found that the prevalence of the −/−K111 genotype in healthy Caucasians individuals was 16.11% (*n* = 180), which stands in stark contrast to healthy African populations (*n* = 127; Table 2), whose prevalence of −/−K111 was 2.36% (*p* = 0.000169). The prevalence of −/−K111 was higher in African American individuals (7.6%; *n* = 39) but this was not statistically significantly different from the prevalence in Caucasian HIV patients and or Caucasian Englishmen. We did not find significant differences in the prevalence of the −/−K111 genotype in populations from Asia or Europe compared to Caucasian Englishmen or Caucasian Americans, except for a slight increase in a population from Siberia (Table 2). A larger sample will be needed in order to determine whether a significant increase in the −/−K111 genotype exists in the Siberian population.

We further evaluated the K111 genotype in a collection of DNA from Caucasian patients with psoriasis, HIV, lupus erythematosus, Takayasu arteritis, breast cancer and leukemia/lymphoma to determine whether the −/−K111 genotype might be associated with other skin or lymphocytic disorders and/or cancers other than CTCL. To study the prevalence of a −/−K111 genotype in a skin disorder other than CTCL, we screened the DNA samples of Caucasian individuals with psoriasis who lived in the Michigan area (Table 3). The prevalence of −/−K111 in these patients was 15.4% (*n* = 123), similar to the prevalence in healthy Caucasians. We did not find differences in the outcome of the −/−K111 genotype in psoriasis patients compared to the +/+K111 genotype as judged by nail involvement, the need for biologic or systemic treatment, the age of onset and/or any unique HLA type.

**Table 3:** Frequency of Detection of −/− K111 in DNA in Caucasian patients with different diseases

Disease	Origin	No. Tested	−/− K111	Frequency of −/− K111 (%)	X^2^	*P* value
*Skin Diseases*						
Psoriasis	Michigan	123	19	15.4	0.0017	ns
*HIV Infection*						
HIV LTNP	MACS	25	5	20	0.3046	ns
	Michigan, New York	11	5	45.5	6.3308	0.0118
Total LTNP		36	11	30.55	4.39 6.691	0.0366 0.0097*
Intermediate	MACS	29	6	20.7	0.4593	ns
	Michigan, New York	3	0	0	0.554	ns
Total Intermediate		32	6	18.75	0.192	ns
Rapid AIDS	MACS	30	3	10	0.6362	ns
	New York, Michigan	17	1	5.9	1.16	ns
Total Rapid AIDS		47	4	8.5	1.526	ns
Unclassified	New York, Michigan, MACS	49	5	10.2	0.896	ns
Total		164	25	15.2	0.009	ns
*Solid tumors*						
Breast Cancer	New York, Michigan	53	2	9.4	1.26	ns
*Lymphomas*						
Hodgkin	Michigan	3	0	0	0.554	ns
Mantle cell	Michigan	6	1	16.5	0.048	ns
Marginal zone	Michigan	5	1	20	0.0699	ns
DLBCL	Michigan	17	1	5.9	0.2806	ns
Follicular	Michigan	23	3	13.0	0.1034	ns
CLL	Michigan	11	2	18.2	0.0506	ns
Total		65	8	12.3	0.406	ns
*Autoimmune Disorders*						
Takayasu	Turkey	30	3	10	0.6362	ns
Lupus	Michigan	38	7	18.4	0.1772	ns

The star indicates a significant difference of the frequency of detection of the −/−K111 genotype between the LTNP and Rapid AIDS patients

We were also interested in studying whether the prevalence of the −/−K111 genotype or *pericentromeric instability* might influence the outcome of another T-cell disorder, notably HIV (Table 3). We screened samples from Caucasian, African American, and Hispanic patients enrolled in the Multicenter AIDS Cohort Study (MACS) and samples from three other institutions using the categories defined above, i.e. LTNPs, Intermediate Progressors, Rapid Progressors, and unclassified.

Overall, the prevalence of the −/−K111 genotype in HIV-infected Caucasians was 15.2% (*n* = 164), similar to healthy Caucasian individuals. Interestingly, the prevalence of the −/−K111 genotype in Caucasian patients with different presentations of HIV infection was 30.55% (*n* = 36) in HIV LTNPs or controllers, 18.75% (*n* = 32) in Intermediate Controllers, and 8.5% (*n* = 47) in Rapid Progressors (Table 3). A statistically significant difference was found in the prevalence of detection of the −/−K111 genotype between the group of HIV LTNPs and all Rapid Progressors (*p* = 0.0097).

We next evaluated the prevalence of the −/−K111 genotype in other cancers as well as in autoimmune disorders. The prevalence of −/−K111 in Caucasian women with breast cancer was 9.4% (*n* = 53) (Table 3) while in African women with breast cancer it was 1.44% (*n* = 139) (Table 4). This difference is likely linked to the ethnicity rather than the disease, as the prevalence of detection of the −/−K111 genotype in African patients with breast cancer is similar to that found in modern healthy African populations of 2.36% (*n* = 127), suggesting again that the −/−K111 genotype originated out of Africa. In Caucasian patients with systemic lupus erythematosus and Takayasu arteritis, the prevalence of the −/−K111 genotype was 18.4% (*n* = 38) and 10% (*n* = 30), respectively (Table 3), similar to the prevalence in healthy Caucasian individuals.

**Table 4:** Frequency of Detection of −/−K111 in DNA from African and African American patients with different diseases

Disease	Origin	No. Tested	−/−K111	Frequency of −/−K111 (%)	*X* ^*2*^	*P value*
African						
Healthy	Nigeria	127	3	2.36		
					14.583	0.00001#
African Breast Cancer	Nigeria	139	2	1.44	0.307	ns
					18.2223	0.00002#
*Total African*		266	5	1.8		0.00001#
HIV/AIDS	Michigan, New York	6	1	16.6	0.216	ns
unclassified	Michigan, New York	5	0	0	0.564	ns
HIV LTNP	Baltimore	18	1	5.6	0.340	ns
DLBCL	Michigan	3	1	33	1.41	ns
CTCL	Michigan	4	0	0	0.452	ns
Breast Cancer	Michigan	3	0	0	0.574	ns
Total African American all diseases		39	3	7.6	1.821	ns

Statistical analysis was determined between healthy individuals and patients of the same ethnicity. # indicates significant differences between healthy Caucasian individuals and healthy African individuals or African patients with breast cancer

### Mapping of centromeric proviruses in CTCL patients

We have shown that K111 is present in 15 different human centromeres [25], with chromosomes 21 and 22 having the highest number of these proviruses. Recently, we described the discovery of a second centromeric HK2 we call K222 that resides in many pericentromeric regions [26]. We were first able to detect this set of K222 pericentromeric proviruses in DNA samples from CTCL cells that have the −/−K111 genotype. K222 has no 5’LTR and has a deletion of *gag* and much of *pro* (Fig. 1a). K222 shares a high homology to K111 in the 3′ end of the provirus. The 3’end of K222 has an LTR, but it lacks the 6 base pair target site duplication specific to K111 and is inserted in a pCER element instead of being inserted in CER:D22Z3 [25, 26].

**Fig. 2:**
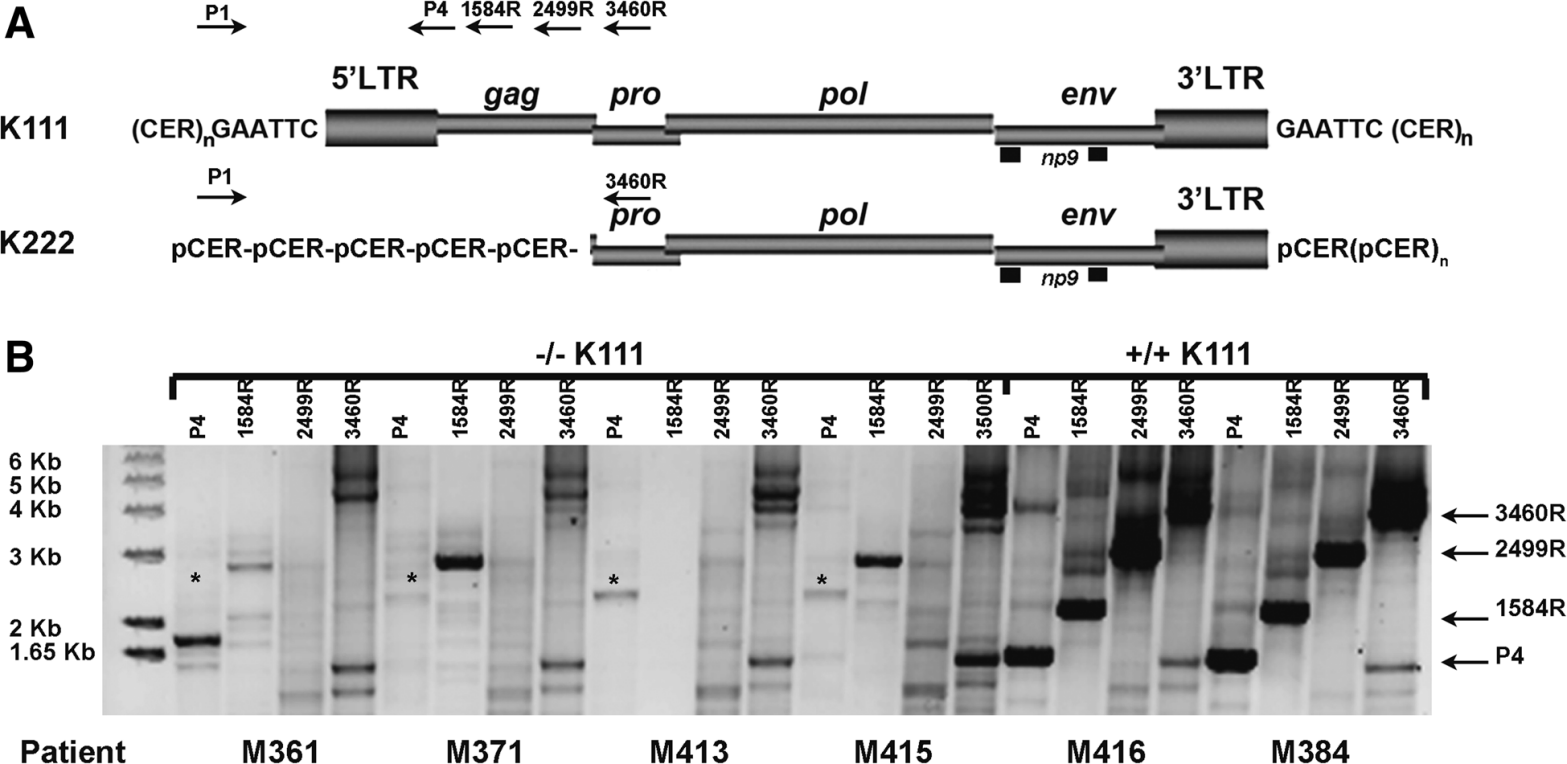
5′ Mapping of pericentromeric HERV-K proviruses in CTCL patients. **a** Schematic representation of the primer sets used to amplify centromeric HERV-K (HML-2) proviruses by PCR. The genomic structure of centromeric HERV-K (HML-2) proviruses K111 and K222 is shown; the viral genes *gag*, *pro*, *pol*, *env* and *np9*, surrounded by LTRs, are integrated into centromeric repeats (CER:D22Z3). At the time of integration, target site duplications are produced at each site of the provirus, which in the case of K111 is GAATTC. The forward primer P1 was used in combination with four reverse primers: P4, 1584, 2499 and 3460 (the numbers correspond to the nucleotide position in reference to K111) that span the HERV-K (HML-2) *gag* and *pro* genes. The product of these primer sets is indicated by the arrows on the right i.e. P4 etc. **b** K222 provirus is detected by PCR in the DNA of the CTCL −/−K111 patients only by the primer set P1–3460 (low molecular weight band shown by the arrow), which was confirmed by sequencing [26]. These DNAs do not produce the expected bands obtained with the 3 reverse primers P4, 1584 and 2499 as shown in the samples from patients with a +/+K111 genotype. The asterisk indicates a band, shown by sequencing, to be a non-specific PCR amplification product of HERV-K (HML-2) 3p25.3

We wanted to assure ourselves that the centromeric proviruses that remain in the DNA of CTCL individuals with the −/−K111 genotype have a provirus pattern similar to the pattern we previously characterized in healthy people. To determine the structure of centromere proviruses, we mapped the DNA of individuals, using a set of primers that specifically amplify centromere K111 and K222 [25, 26]. We found that in the DNA of CTCL patients with the −/−K111 genotype, no K111 beta band (mw 1614 bp) was seen by PCR (Fig. 2) using the primer pair P1 and P4 as also shown in Fig. 1. Similarly, no amplification products were seen in the same −/−K111 patients when the DNA was amplified with reverse primers that bind at bases 1584 in *gag* and 2499 in *pro,* areas which are absent in the K222 genome (Fig. 2). Amplification products with these primers were readily seen in an individual with a +/+ K111 genotype. Using the primer that binds to 3460 of the *pro* region of both K111 and K222, a low molecular weight band was barely visible in the −/−K111 patients, while a strong band at 4240 bp was seen in +/+K111 normal patients. The low molecular weight band is from K222, which can be detected in both −/− and +/+K111 individuals. These results indicate that the pattern of genomic composition of centromere proviruses in −/−K111 is the same in all the CTCL patients studied, as we had previously determined in CTCL cell lines [25].

### Quantitation of K111 and K222 in humans

**Fig. 3:**
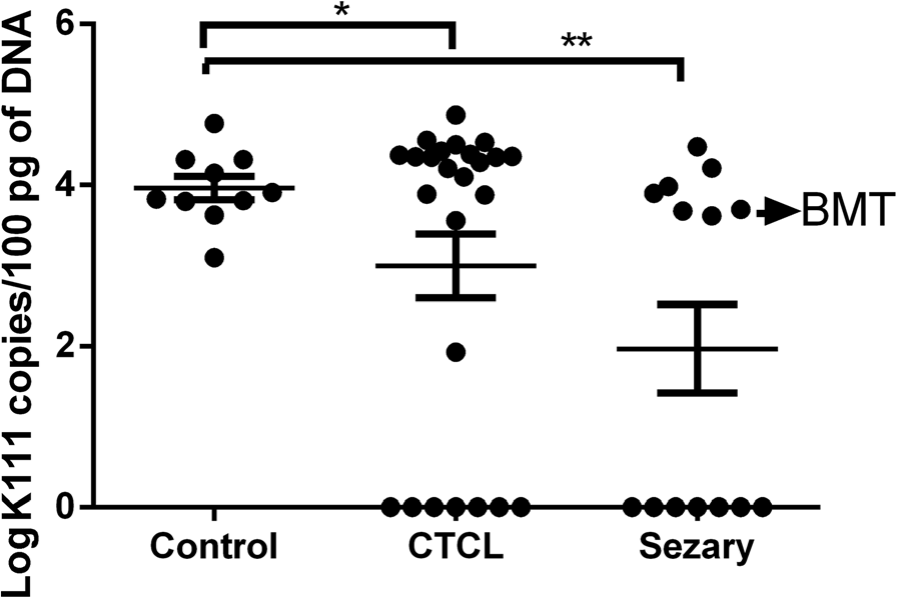
Copy number of centromeric K111 in different stages of CTCL. Centromeric K111 was quantitated in the DNA of PBLs by qPCR using a LNA primer that is highly specific for K111 *gag*. The copy number was determined in the same run using a standard curve generated by dilution of DNA chosen from a CTCL patient who had the highest levels of K111 measured in our earliest assays. Note that one CTCL patient had a relatively low copy number while all the rest of the patients have either high copy numbers of K111 or have a −/−K111 genotype. Note that the DNA from the PBLs of a patient who had a bone marrow transplant (BMT) had a high copy number of K111 while her buccal swab (Fig. 1c) is −/− K111. *p* values were calculated using a t-test. Statistical significance *p* < 0.05 (*), *p* < 0.01 (**)

We were interested in trying to gauge how many copies of the K111 and K222 proviruses might be in a person’s genome. This was important in order to elucidate if +/+K111 patients with CTCL may have reduced numbers of K111. We developed an assay using primers modified with locked nucleic acids (LNA), which bind with enhanced affinity to the target K111 sequence in the *gag* region of K111, but not to K222 or other HK2 proviruses [25]. Using this assay, we screened DNA from all of our CTCL patients, which yielded the results shown in Fig. 3. Patients with CTCL and, the severe form Sz, had statistically significantly fewer copy numbers of K111 than did control subjects. All −/−K111 patients detected with the P1 and P4 primers (Figs. 1 and 2, Table 1) had an undetectable proviral load in their genome using the LNA primer assay system. The inability to detect K111 with the LNA primer set in the *gag* region of −/− K111 individuals also indicates that the failure to detect K111 is not due to some mutation in the CER elements flanking the virus, but is due to the absence of K111. The K111 LNA assay detected numbers of K111 copies in the human population that varied within a range of 1230 to 58,000 copies/100 pg of DNA (average 11,220 copies/100 pg of DNA), which corresponds to 78 to 3480 copies/genome in somatic cells. These numbers of proviruses are similar to the ones previously estimated in our laboratory [25]. We did not find statistically significant differences in the number of copies of K111 in +/+K111 individuals who are healthy or in those who have CTCL. Only one CTCL patient had 85 copies of K111 proviruses/100 pg of DNA (Fig. 3). Nonetheless, the −/−K111 genotype was readily evaluated with the LNA technology and gave no amplification products. This suggests that any association of K111 copies with severe CTCL disease and Sz is determined by having a −/−K111 genotype rather than fewer copies of K111.

**Fig. 4:**
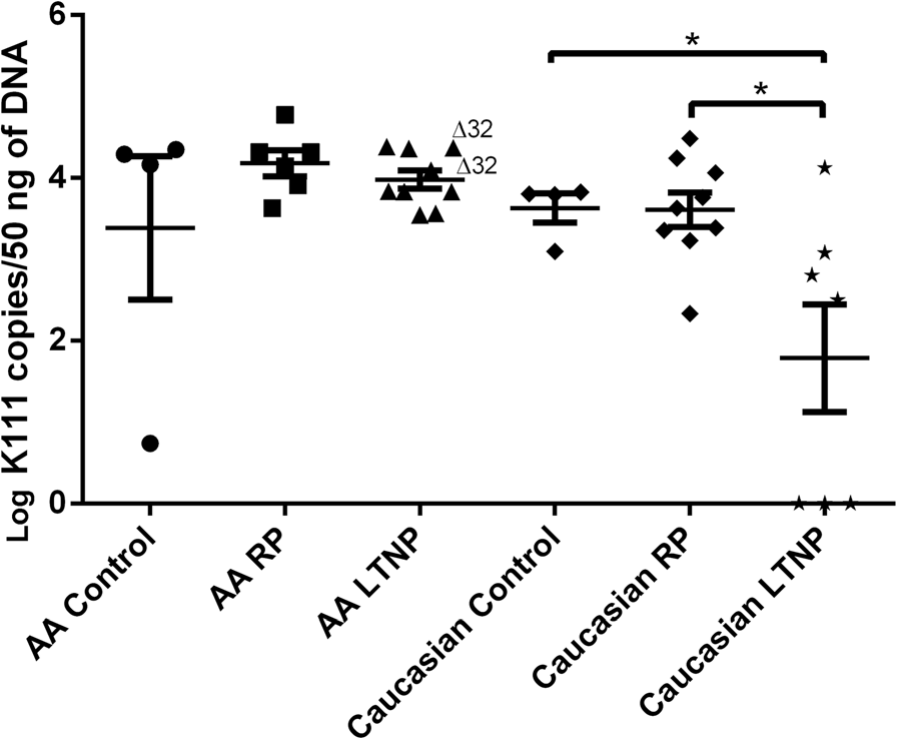
Copy number of centromeric K111 in different stages of HIV progression. K111 proviral copy number was quantitated in DNA from patients with HIV using qPCR with the LNA primer specific for K111. AA RP or Caucasian RP represents African American or Caucasian patients with HIV who are HIV Rapid Progressors (RP). Note that the copy number of the Caucasian Long-Term Non-Progressors (LTNPs) was significantly lower than in Caucasian controls and RPs. *p* values were calculated using a t-test. Statistical significance *p* < 0.05 (*), *p* < 0.01 (**)

We then determined the numbers of K111 proviruses in HIV-1 infected people in order to investigate whether HIV LTNPs have fewer copies of K111 proviruses than HIV Intermediate and/or HIV Rapid Progressors (Fig. 4). Quantitation of K111 copies using our K111 LNA assay revealed that in contrast to HIV negative subjects and to all Rapid Progressors of Caucasian ethnicity, the copy number of K111 decreases significantly in HIV LTNPs. However, we did not observe this difference between HIV LTNPs of African American ethnicity as compared to their ethnic controls or African American HIV Rapid Progressors, confirming the observations noted above (Fig. 4).

**Fig. 5:**
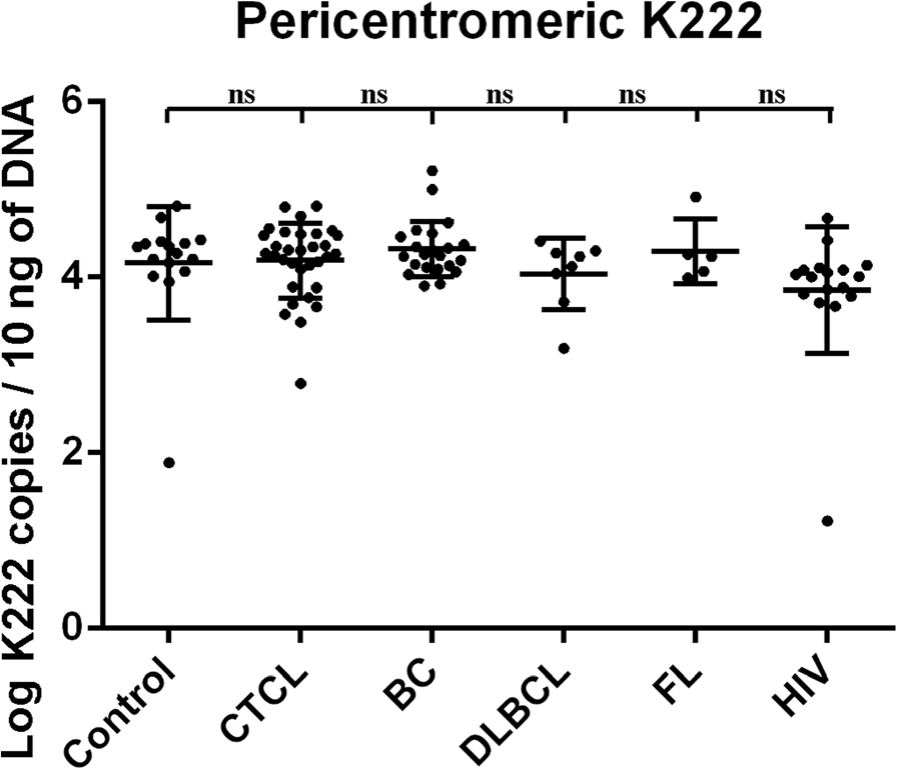
Copy number of pericentromeric K222 in cancer, HIV, and control subjects. The proviral copy number of K222 was quantitated by qPCR using the primers K222F and K222R, and the molecular probe K222P, which specifically detect K222 and no other HERV-K proviruses. The copy number of K222 was calculated in the DNA of the PBLs of control subjects, patients with CTCL, breast cancer (BC), diffuse large B cell lymphoma (DLBCL), follicular lymphoma (FL), and HIV. The copy number is an estimate of the approximate number of copies per genome and was normalized to the levels of *gapdh* as described in the methods section. No significant differences were observed in the mean copy number of K222 in cancer or HIV patients as compared to the control subjects. ns = not significant

Having determined that human populations of different ethnicities have two different genotypes of centromeric K111 proviruses, +/+K111 or −/−K111, we looked at centromeric K222 proviruses, which also have multiple copy numbers in the pericentromeric regions, to evaluate whether there is any difference in the numbers of K222 proviruses in CTCL or HIV populations. We estimated the numbers of K222 centromeric proviruses that exist in 9 human chromosomes to be approximately 8 to 80 copies using an assay we previously described [26]. Quantitation of K222 revealed that the number of K222 proviruses does not change significantly between control subjects, patients with CTCL or other cancers, or HIV (Fig. 5). While there was variation in the copy number of K222 in diverse human populations in this study, no significant changes were seen in any of the disease subsets as compared to controls.

### Genetic variability of K111 proviruses in the human genome

K111 s exist in the centromeres in up to 15 different chromosomes, but are mostly found in chromosomes 21 and 22. As K111 s appear to have spread via homologous recombination in the centromeres at different times during the evolution of modern humans, one would expect to see genetic variation of K111 in chromosomes 21 and 22. We sequenced the K111 PCR fragments amplified from DNA from human/hamster hybrid cell lines containing human chromosomes 21 or 22 and also DNA from patients with CTCL and HIV using the PacBio platform that can sequence single DNA fragments up to 15–20,000 bp. This approach assures that all sequences present in a PCR fragment represent one of the many K111 proviruses in that PCR product.

**Fig. 6:**
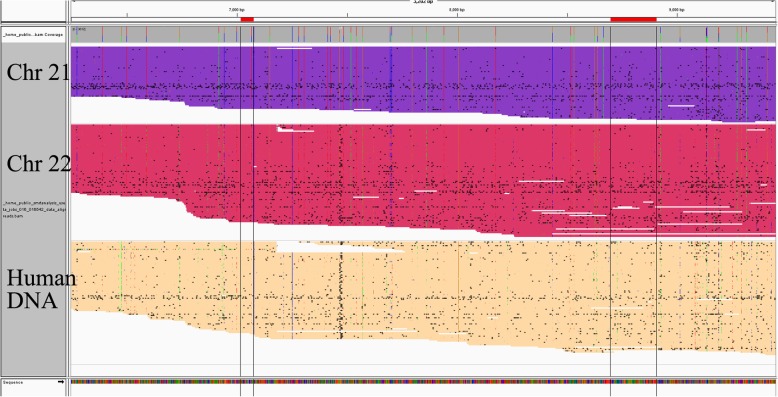
PacBio Sequencing of centromeric K111 in Chr 21, 22 and Human DNA. A squished plot of the PacBio sequences generated from the 3′ side of K111 in the hamster/human chromosome 21 and 22 hybrids, as well as normal human DNA. The squished map only shows indels (black dots) that represent subsets of K111 proviruses that are different from the deposited K111 sequence. Numerous shared indels can be seen in many of the sequences, suggestive of the presence of hundreds of unique K111 proviruses in these chromosomes. There appear to be some unique sequences in chromosome 21 compared to chromosome 22. The squished map of sequences amplified from human DNA display sequence patterns similar to those found in Chr 21 and 22

DNA sequence analysis revealed that the K111 proviruses have accumulated many sequence mutations over time (Fig. 6), with many unique to chromosome 21 or 22. The same K111 sequences seen in these hybrid cells were also present in DNA isolated from human DNA samples. This genetic variability is not some type of recombination that arose in the hybridization of these human chromosomes within hamster cells, as the hamster genome lacks HK2 proviruses, and as similar sequences were found in human DNA. The sequences that were seen had many shared indels. The numerous indels variations of K111 s appear to have arisen by homologous recombination over evolutionary time, confirming our previous observations [24–26].

### Genetic variability of K111 in CTCL and HIV patients

**Fig. 7:**
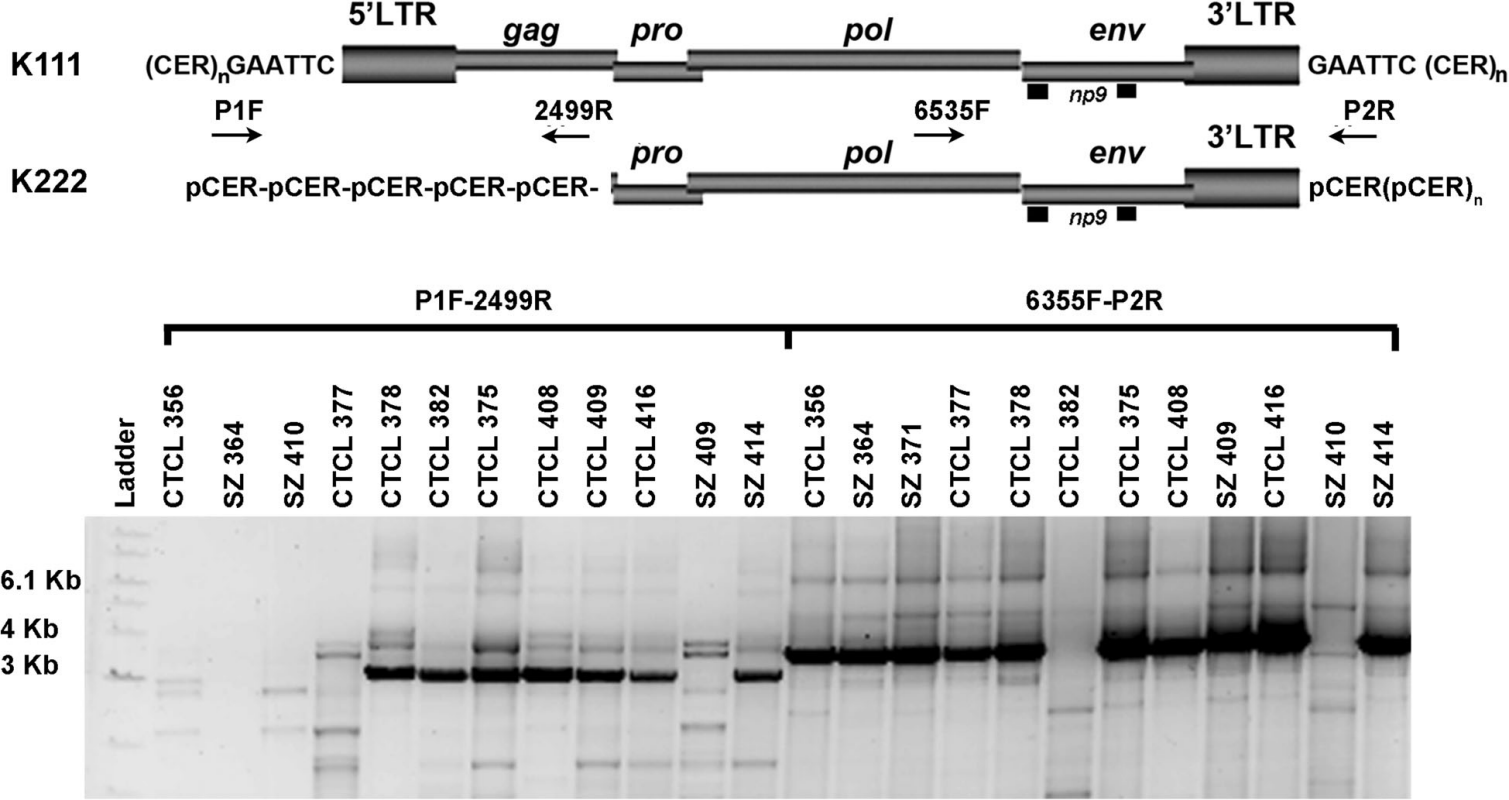
Barcoding of Amplicons of K111 in CTCL patients. The diagram depicts the 5′ portion of K111 that was amplified with primers P1F and 2499R, or the 3’portion of K111 amplified with primers 6535F and P2R. DNA samples were bar-coded so that they could be identified when these PCR amplicons were used for deep sequencing using the PacBio technology as described in Materials and Methods. Strong bands between 3 Kb to 4 Kb were cut from gels and purified for deep-sequencing analysis. The −/− K111 genotype did not provide an amplicon with P1F and 2499R. Two samples that did not amplify using the primers 6355F and P2R had likely interference with the barcode used. Using a different barcode, these samples produced the right amplification products (data not shown)

To determine the genetic variability of K111 sequences in CTCL and HIV patients that have either a +/+ or −/−K111 genotype, we performed PacBio Next-Generation-Sequencing of K111 PCR products generated with primer P2R that binds the 3′ integration site of K111 CER:D22Z3, and primer 6353F that binds the *env* region of K111. We also amplified another segment using primer P1 that binds the 5′ CER:D22Z3, and primer 2499R that binds downstream of the origin of K111 sequence, in the *pro* region (Fig. 7). Six bp barcodes were added to the 5′ end of 6353F and to the 3′ end of 2499R in order to distinguish between the sequence results from different patients.

A squished plot alignment of the K111 sequences amplified, displaying only indels, revealed a large degree of sequence variability of K111 proviruses amplified in each human DNA sample (Additional file 3: Figure S1). In patients with a +/+K111 genotype, about 200 K111 proviruses with unique sequences were detected. DNA sequencing also confirmed the lack of K111 proviral sequences on the 5′ side of the K111 provirus in −/−K111 samples. In these patients, we were able to amplify a fragment of the 3′ portion of the centromere provirus, which may be related to the pericentromeric K222 proviruses, or recombinant K111/K222 proviruses, as previously reported [25]. Although, as expected, we did not obtain DNA sequences at the 5′ site of the K111 sequence in −/−K111 samples, we did recover non-specific amplification products of the HK2 provirus 3p25.3 (Additional file 3: Figure S1), validating the observations shown in Fig. 1.

Looking at the sequences of 3p25.3 from 4 CTCL individuals generated by our sequencing technology, we found that only two copies of this provirus existed in these individuals per diploid genome, validating the fidelity of the PacBio sequencing. In contrast to K111 sequences that have many indels, we did not find indels in HK2 3p25.3 sequences, but only sporadic mutations that appear to be SNPs representative of each individual (Additional file 4: Figure S2). This supports the idea that the multiple mutations in K111 s were not due to PCR and sequencing artifact, but rather were due to the evolution and spread of K111 throughout the pericentromeric area by a process of homologous recombination [24–26]. DNA sequencing readily validated the existence of −/− and +/+ K111 genotypes.

## Discussion

In this study we show that approximately 15% of Caucasian people lack K111 proviruses in their pericentromeric/centromeric region. In contrast, the −/−K111 genotype was rarely seen in individuals from Nigeria and was not detected in any other sub-Saharan Africans or Pygmy populations, suggesting that the −/−K111 genotype appeared after ancient humans migrated out of Africa [46]. The finding of a −/−K111genotype in 7.6% of African Americans probably arose through the forcible translocation of Africans through the slave trade and then subsequent intermingling with Caucasian and Amerindian populations over many generations. The questions arise: Where did the −/−K111 genotype originate, and how can 15% of Caucasians lack several hundreds to thousands of copies of these pericentromeric proviruses while almost, if not all, African individuals have K111 proviruses?.

In a retrospective study of DNA samples obtained from Neanderthal and Denisovan, late Pleistocene Hominins that moved out of Africa between 50,000 to 400,000 years ago, we found a few copies of K111 in their genomes, suggesting that they already carried K111 out of Africa [25, 28–31]. Thus, the −/−K111 genotype we have detected in modern humans does not appear to come from Neanderthals or Denisovan. It is possible that the −/−K111 genotype was transmitted to early modern humans out of Africa from other primitive hominins that lacked K111. However, it is more likely that some early human who had a +/+K111 genotype in their genome at some point in evolutionary time may have lost the 5′ end of K111 through recombination with K222, a centromere provirus that also lacks the exact 5′ end [26]. We have found evidence of recombinant K111/K222 provirus sequences in human genomes using both PCR-sequencing and Pac Bio Next-Generation-Sequencing studies, suggesting the possibility that K222 recombination with K111 can delete the 5′ domain of K111. These individuals could have evolved into carrying the −/−K111 to different parts of the world. The evidence suggests that this event did not take place in Africa. The worldwide distribution of the −/−K111 is of great interest, and it is possible that in some isolated populations we might find many individuals that exclusively have the −/−K111 genotype.

It is of great interest that so many K111 proviruses exist in the centromeric and pericentromeric area of the human genome. These K111 s significantly outnumber the proviruses inserted into non-centromeric regions. They appear to have expanded to such numbers by homologous recombination, a mechanism that created many indels in K111 proviruses. In addition, there appear to be uniquely different K111 s in chromosome 21 and 22 which have the bulk of the K111 s in our genome. How these proviral differences play a role in disorders of chromosome 21 such as trisomy 21 and/or disorders of chromosome 22 is now under investigation. In the genetic disorder trisomy 21, we have shown that K111 is reduced, with some evidence of further recombination events occurring in these patients’ cells [47].

It should be pointed out that a large number of centromeric proviruses are not unique to man. In fact, kangaroo species have several endogenous retroviruses (KERV) in centromeres found in tandem, and are major integral parts of active centromeres similar to what is seen with human K111 s [48].

We asked the question about diseases that could be associated with the −/−K111 genotype, which produces *pericentromeric instability*. Our studies show no differences in the prevalence of the −/−K111 genotype in Caucasian patients with psoriasis, lupus, Takayasu arteritis, breast cancer and/or lymphomas. However, the prevalence of the −/−K111 genotype was significantly higher in Caucasian individuals with CTCL. These CTCL patients with a −/−K111 genotype were more severely affected by complications like Sz and LCT and more aggressive forms of disease requiring extensive treatment to control the disease. It is possible then that *pericentromeric instability* in Caucasians in some way contributes to pathogenesis of CTCL in these patients, and that missing pericentromeric K111 makes the disease more severe and difficult to control. Therefore, the −/−K111 genotype appears to be an independent risk factor in determining treatment outcome for patients with CTCL. This could be assessed by a prospective study of patients with CTCL in order to understand whether there are outcome differences in disease in −/−K111 vs. +/+K111 patients. Although African and/or African-American patients can develop severe CTCL disease, as do Caucasians, a −/−K111 genotype is less prevalent. We have insufficient data to understand the role of −/−K111 in African American patients. Only 4 patients in our cohort were African-Americans and 2 had severe disease; both had a +/+K111 genotype. When considering the role of centromeric K111 s in human disease, as we have shown, these proviruses may serve as a marker of the health of the pericentromeric genome [47].

It has been reported that in patients with CTCL there is overexpression of HERV-K and HERV-W mRNA compared to healthy individuals, but there was no difference in expression levels in more advanced disease [49]. We did not assay the extent of expression of K111 s’ putative gene *np9*, or spliced variants of this gene [32, 33], and/or other genes of K111, but the role of expression of these genes in CTCL is of great interest for future studies.

In some cases, patients with severe CTCL may require bone marrow transplantation. It is possible that donors with a +/+K111 genotype may be more beneficial than a −/−K111 donor. This could be ascertained by retrospective studies of outcome in such transplanted patients where −/−K111 genotyping of donors could be performed. One of our −/−K111 patients received a +/+K111 genotype donor marrow. She had engraftment with good control of her disease. Unfortunately, she subsequently died of cardiac complications from earlier chemotherapy. Screening donors for a +/+K111 genotype might result in better control of disease in these sick patients.

As a group, Caucasian HIV patients have a 15.3% prevalence of the −/−K111 genotype. However, there is a statistically significant higher prevalence of −/−K111 genotype in Caucasian/Hispanic patients who are LTNPs as compared to Rapid Progressors. It is conceivable that the numbers of centromeric K111 copies in the genome increases the risk factor of AIDS development, while missing centromeric K111 can add to better control of HIV. The low prevalence of −/−K111 in one population vs. high prevalence of −/−K111 in another population might also contribute to the differences in rates of spread of HIV in the world with −/−K111 acting as limiting factor in transmission. Larger studies will be necessary to confirm these results.

We should note that K111 was first discovered in the blood of HIV infected individuals, the only type of individuals in whose blood we have found RNA transcripts of these centromeric proviruses [24, 25]. K111 RNA was not detected in the blood of healthy individuals or patients with breast cancer or several types of lymphomas. We previously observed that HIV upregulates K111 via Tat protein transactivation of the K111 promoter. In addition, Tat was found to relax the heterochromatin at centromeric areas, allowing for transcriptional activation of K111 sequences [50, 51]. It would be interesting to study whether the transcriptional activation of K111 affects HIV pathology and disease progression, and whether not having K111 negatively affects the course of HIV infection. It is of interest that the −/−K111 cell lines Hut 78 and H9, which have the CD4 receptor for HIV, support the growth of HIV, producing high titers of virus without going through cell lysis. These cell lines originated from a patient with CTCL and produced sufficient virus to make a successful HIV test for ELISA and Western Blotting [37, 52]. These cell lines do not need to be replenished with feeder cells, but continuously produce significant titers of HIV and do not undergo cell death. How these cells resist cell lysis and death remains unexplained, but perhaps the −/−K111 genotype plays a role in preventing cellular death.

This study adds to our understanding of how HK2 viruses may play a role in control of HIV infection. It has been shown that HK2 *gag* interacts with HIV *gag* reducing HIV-1 release and infectivity, thus interfering with HIV replication [53, 54]. In addition, HIV-1 induces aberrant expression of HK2 Env [55]. This allows human T-cell clones to HK2 and human anti HK2 Env antibodies to eliminate HIV infected cells in vitro [56–59]. In addition, elite controllers were shown to have strong HK2 antibody as well as cellular responses to HK2 providing protection against aggressive viral replication [55].

Finally, K111 s are type 1 proviruses that have the ability to make functional Np9 proteins, similar to other HK2 Type 1 proviruses. Recent evidence has shown that K111 can also produce alternative spliced variants of *np9* [32]. The function of Np9 has begun to be elucidated, but the function of Np9 variants is not yet known. Therefore, K111 encoded Np9 protein variants may play some important role in T-cell biology and HIV infection. In the case of CTCL, the absence of K111 *np9* variants may contribute to failure of cells to die and thus to become neoplastic, while in HIV this may provide protection against T-cell lysis from HIV and result in better preservation of T-cells as seen in HIV LTNPs. Clearly, modern humans have hundreds to thousands of copies of K111, each one with the potential of generating an Np9 protein or variants of Np9. Greater understanding of these proteins may be very important for delineating the function of the T-cell in CTCL and/or in HIV infection, leading perhaps to better therapies for T-cell disorders.

## Conclusions

Our data indicate that HK2 proviruses in the pericentromeres of several chromosomes, or at least the 5′ portion of their genomes, are deleted in a subset of modern humans. Considering that ~ 1000 of these retroelements exist in the pericentromeres, having a deletion in the 5′ portion of these retroelements indicates that 3400 Kb of pericentromeric sequence is missing. Further, it appears that *pericentromeric instability* is associated with survival of a subset of CD4+ T-cells, which together contribute to the development of neoplastic transformation with concomitant severe CTCL. In another unknown way, pericentromeric instability allows a different subset of CD4+ T-cells to survive better in HIV infection resulting in long term survival.

## Additional files

Additional file 1: **Table S1.** Clinical Features of Cutaneous T-cell Lymphoma patients: age, gender, mortality, diagnosis, disease transformation, and the development of secondary cancers in patients with CTCL (DOCX 16 kb)

Additional file 2: **Table S2.** Treatment of Patients with Cutaneous T-Cell Lymphoma (DOCX 14 kb)

Additional file 3: **Figure S1.** Squished plot of K111 PacBio sequences from patients with CTCL**.** The squished maps show several K111 sequences seen among the patients, with as many as 200 different genetic patterns visible. Shown are sequences amplified in individuals with a −/−K111 or +/+K111 genotypes (see Y axis). Each color plot represents an individual patient. Differences in nucleotide sequences are indicated by black dots. These black dots only represent indels. Single nucleotide polymorphisms (SNPs) are not displayed for clarity. Note that in −/−K111 patients a cluster of sequences on the 5′ site, but not the 3′ site, show the HERV-K HML-2 provirus 3p25.3. Black dots in 3p25.3 sequences only indicate indels relative to the 5′ K111 sequence. No particular pattern of K111 could be recognized that distinguished CTCL patients from Sézary patients other than the absence of K111 sequences on the 5’side of K111 (PDF 6634 kb)

Additional file 4: **Figure S2.** Highlight plot of HERV-K HML-2 3p25.3 sequences. The highlight plot displays the differences in nucleotide sequence of the provirus 3p25.3 amplified by PCR and sequenced using the PacBio platform. Sequences are compared to provirus 3p25.3 (Acc No. JN675020.1), the master sequence. The plot indicates nucleotide substitutions T (red ticks), A (green ticks), C (blue ticks), and G (yellow ticks). A neighbor joining phylogenetic tree is indicated at the right side. The highlight plot was generated using Highlighter from Los Alamos HIV Sequence Database https://www.hiv.lanl.gov/content/sequence/HIGHLIGHT/highlighter_top.html. This indicates that variation in fixed endogenous viral sequence occurs mostly by spontaneous mutations over time rather than homologous recombination which is so characteristic of the enormous sequence variation seen in K111 (PDF 3482 kb)

## Abbreviations

CLL: Chronic lymphocytic leukemia
CTCL: Cutaneous T-cell Lymphoma
HK2: Human Endogenous Retroviruses type K HML-2
LCT: Large cell transformation
LNX: Ligand of numb protein X
LTNP: Long-Term Non-Progressors
LTR: Long Terminal Repeat
MACS: Multicenter AIDS Cohort Study
PBLs: Peripheral blood lymphocytes
PBMCs: Peripheral blood mononuclear cells
PLZF: Promyelocytic leukemia zinc finger protein
Sz: Sézary syndrome
TCR: T-cell receptor
