# Supplementary material for: Structural variation of centromeric endogenous retroviruses in human populations and their impact on cutaneous T-cell lymphoma, Sézary syndrome, and HIV infection
Source: BMC Med Genomics. 2019 May 2;12:58. doi: 10.1186/s12920-019-0505-8 (PMC6498702; doi:10.1186/s12920-019-0505-8)
Supplement: Supplementary file 1 — Table S1. Clinical Features of Cutaneous T-cell Lymphoma patients: age, gender, mortality, diagnosis, disease transformation, and the development of secondary cancers in patients with CTCL (DOCX 16 kb) [file 12920_2019_505_MOESM1_ESM.docx]

**S1 Table.** Clinical Features of Cutaneous T-cell Lymphoma patients: age, gender, mortality, diagnosis, disease transformation, and the development of secondary cancers in patients with CTCL.

| Clinical Attributes | -/- K111 | +/+ K111 |
| --- | --- | --- |
| Total number of patients | 13 | 26 |
| Average age | 66.2 | 61.4 |
| Male/female | 8/5 | 16/10 |
| Deceased | 0 | 9 (34.6%) |
| Sèzary Syndrome | 5 (41.6%) | 7 (29.2%) |
| Large Cell Transformation | 4 | 7 |
| Second primary cancer | | |
| Hodgkin Lymphoma | 2 | 0 |
| Peripheral T-cell Lymphoma | 2 | 1 |
| Chronic Lymphocytic Leukemia | 0 | 2 |
| Lung Cancer | 1 | 0 |
| Esophagus Cancer | 0 | 1 |
| Prostate Cancer | 1 | 1 |
| Mesothelioma | 0 | 1 |
| Melanoma | 2 | 4 |
| Squamous cell Carcinoma | 2 (1 died of metastatic disease) | 2 |
| Basal Cell Carcinoma  Total # of patients with Second primary cancer  Total # of patients with 1 or more Cancers | 2 | 3 |
|  | 11/13  4/13 | 17/26  6/24 |
| CCR5 Δ‎32 mutation | 3/11=27.3% | 5/23=23.7% |
