# Supplementary material for: Structural variation of centromeric endogenous retroviruses in human populations and their impact on cutaneous T-cell lymphoma, Sézary syndrome, and HIV infection
Source: BMC Med Genomics. 2019 May 2;12:58. doi: 10.1186/s12920-019-0505-8 (PMC6498702; doi:10.1186/s12920-019-0505-8)
Supplement: Supplementary file 2 — Table S2. Treatment of Patients with Cutaneous T-Cell Lymphoma (DOCX 14 kb) [file 12920_2019_505_MOESM2_ESM.docx]

**S2 Table.** Treatment of Patients with Cutaneous T-Cell Lymphoma.

| Treatment | -/- K111 (n=13) | +/+ K111(n=26) |
| --- | --- | --- |
|  |  |  |
| Bexarotene (tagretin) | 7 | 20 |
| Acitretin (retinoid) | 2 | 7 |
| Interferon alpha | 6 | 15 |
| Interferon gamma | 1 | 1 |
| Romidepsin (HDAC inhibitor) | 3 | 7 |
| Vorinostat (HDAC inhibitor) | 6 | 11 |
| Gemcitabine | 1 | 2 |
| Pralatrexate (folate inhibitor) | 3 | 7 |
| Methotrexate (folate inhibitor) | 3 | 1 |
| Brentuximab (anti-CD30) | 6 | 6 |
| Denileukin diftitox (IL2-diphtheria toxin) | 1 | 4 |
| Alemtuzumab (anti-CD52) | 1 | 4 |
| Hydroxydaunorubicin (anthracycline antibiotic) | 1 | 4 |
| PEP-C* | 1 | 8 |
| Narrow band UV treatment | 5 | 18 |
| PUVA** | 3 | 10 |
| Extra corporeal phototherapy | 2 | 6 |
| Radiation to skin (*p* value : 0.014) | 4 (18 treatments) | 15 (49 treatments) |
| Radiation to total body | 4 | 5 |
| Bone Marrow Transplant | 2 | 3 |
| Topical nitrogen mustard therapy | 2 | 4 |
| Other | 2 | 1 |

Shown are the treatments that patients received for their CTCL over many years of therapy. The intense treatments needed to control disease, indicates how these patients probably differ from most patients with CTCL. *daily prednisone 20mg, cyclophosphamide 50mg, etoposide 50mg, procarbazine 50mg daily orally. **psoralen and ultraviolet A light therapy.
