# Supplementary figures and images for: Structural variation of centromeric endogenous retroviruses in human populations and their impact on cutaneous T-cell lymphoma, Sézary syndrome, and HIV infection
Source: BMC Med Genomics. 2019 May 2;12:58. doi: 10.1186/s12920-019-0505-8 (PMC6498702; doi:10.1186/s12920-019-0505-8)

3p25.3

+/- K111

-/- K111

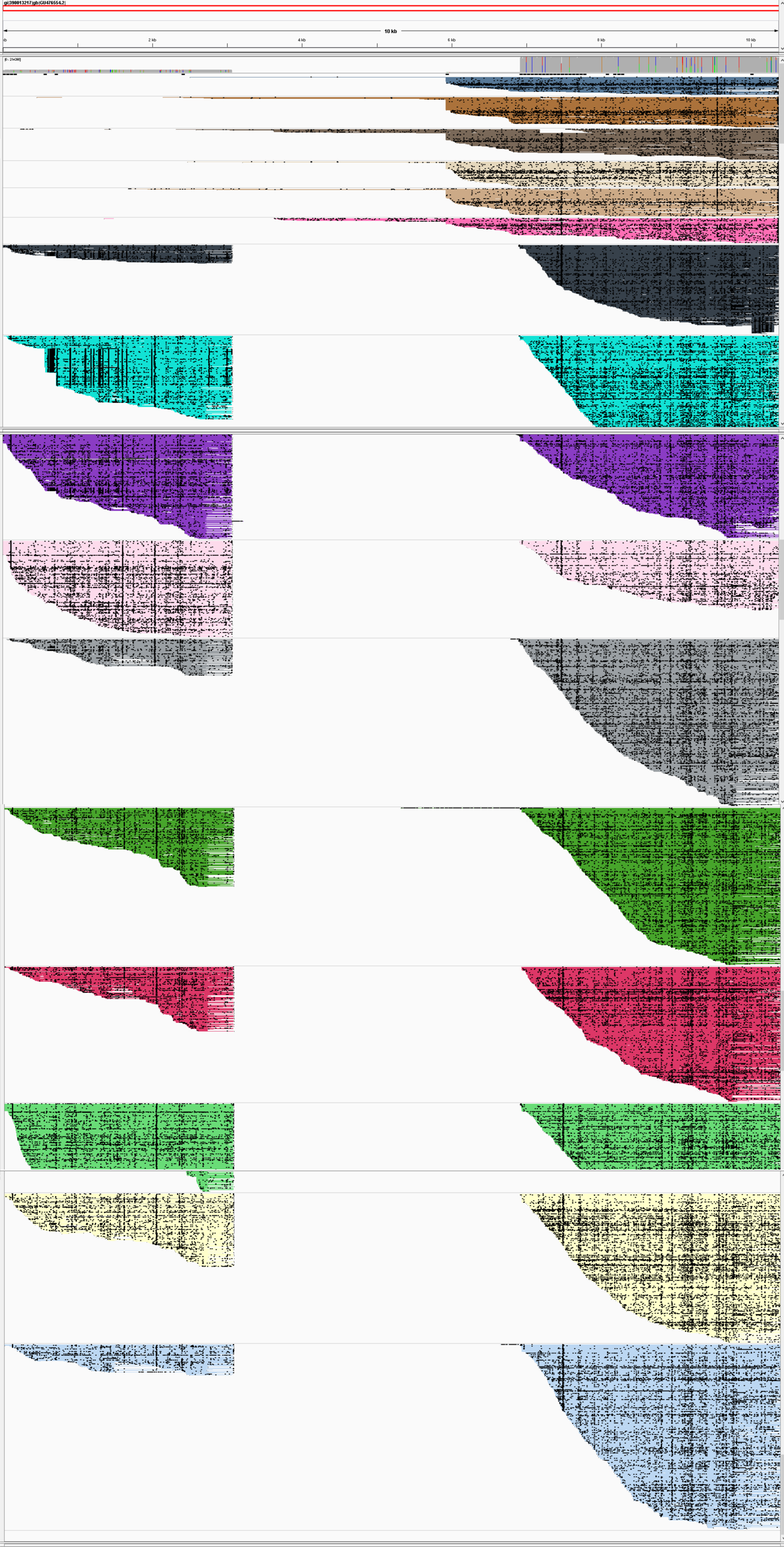

Supplement: Supplementary file 3 — Figure S1. Squished plot of K111 PacBio sequences from patients with CTCL. The squished maps show several K111 sequences seen among the patients, with as many as 200 different genetic patterns visible. Shown are sequences amplified in individuals with a −/−K111 or +/+K111 genotypes (see Y axis). Each color plot represents an individual patient. Differences in nucleotide sequences are indicated by black dots. These black dots only represent indels. Single nucleotide polymorphisms (SNPs) are not displayed for clarity. Note that in −/−K111 patients a cluster of sequences on the 5′ site, but not the 3′ site, show the HERV-K HML-2 provirus 3p25.3. Black dots in 3p25.3 sequences only indicate indels relative to the 5′ K111 sequence. No particular pattern of K111 could be recognized that distinguished CTCL patients from Sézary patients other than the absence of K111 sequences on the 5’side of K111 (PDF 6634 kb) [file 12920_2019_505_MOESM3_ESM.pdf]

Mismatches compared to master

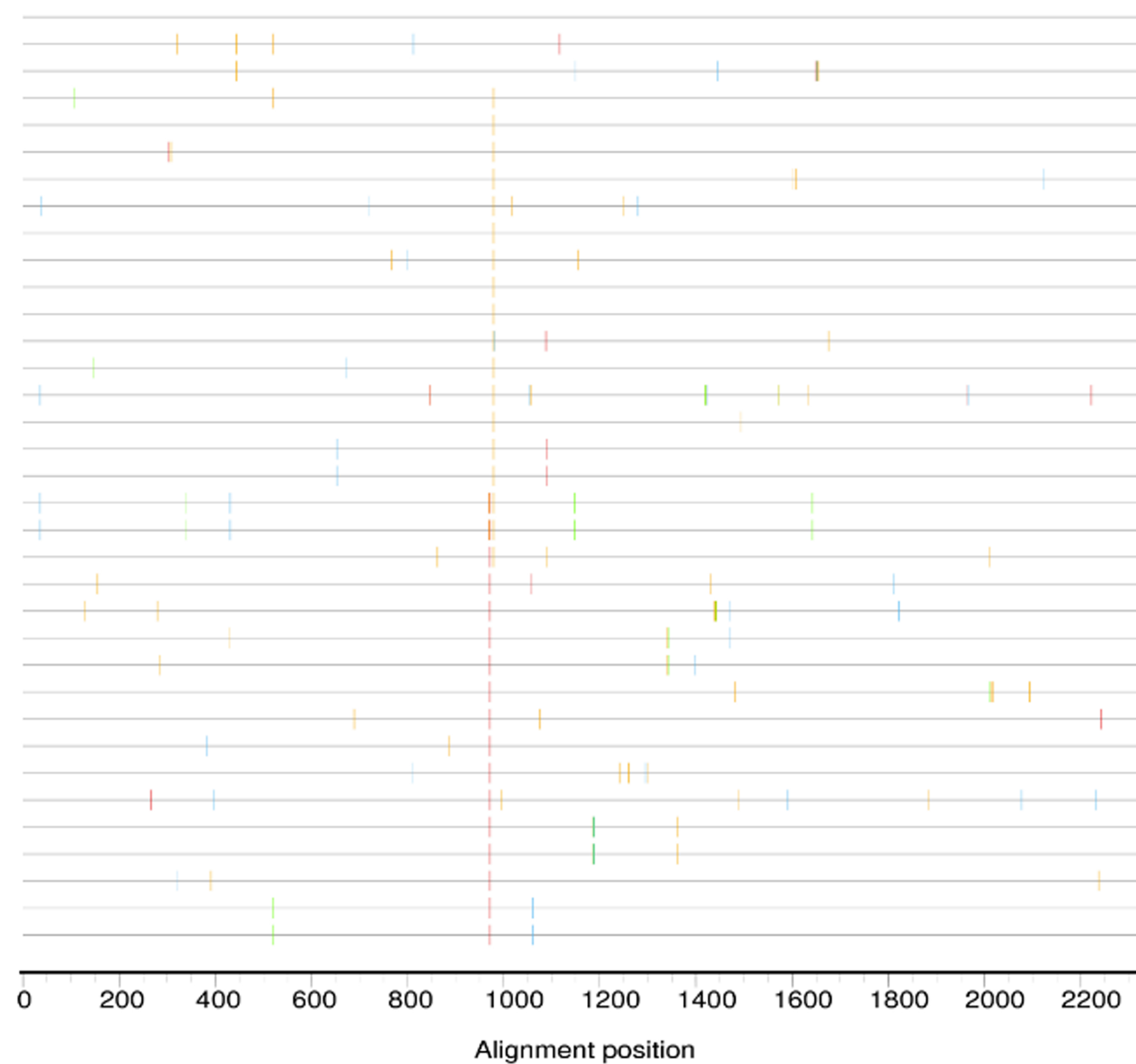

3p25.3 (m)

1  
24  
37  
38  
9  
10  
14  
6  
32  
36  
35  
2  
23  
22  
13  
16  
18  
19  
20  
11  
12  
4  
29  
27  
17  
8  
28  
30  
31  
33  
34  
7  
25  
26

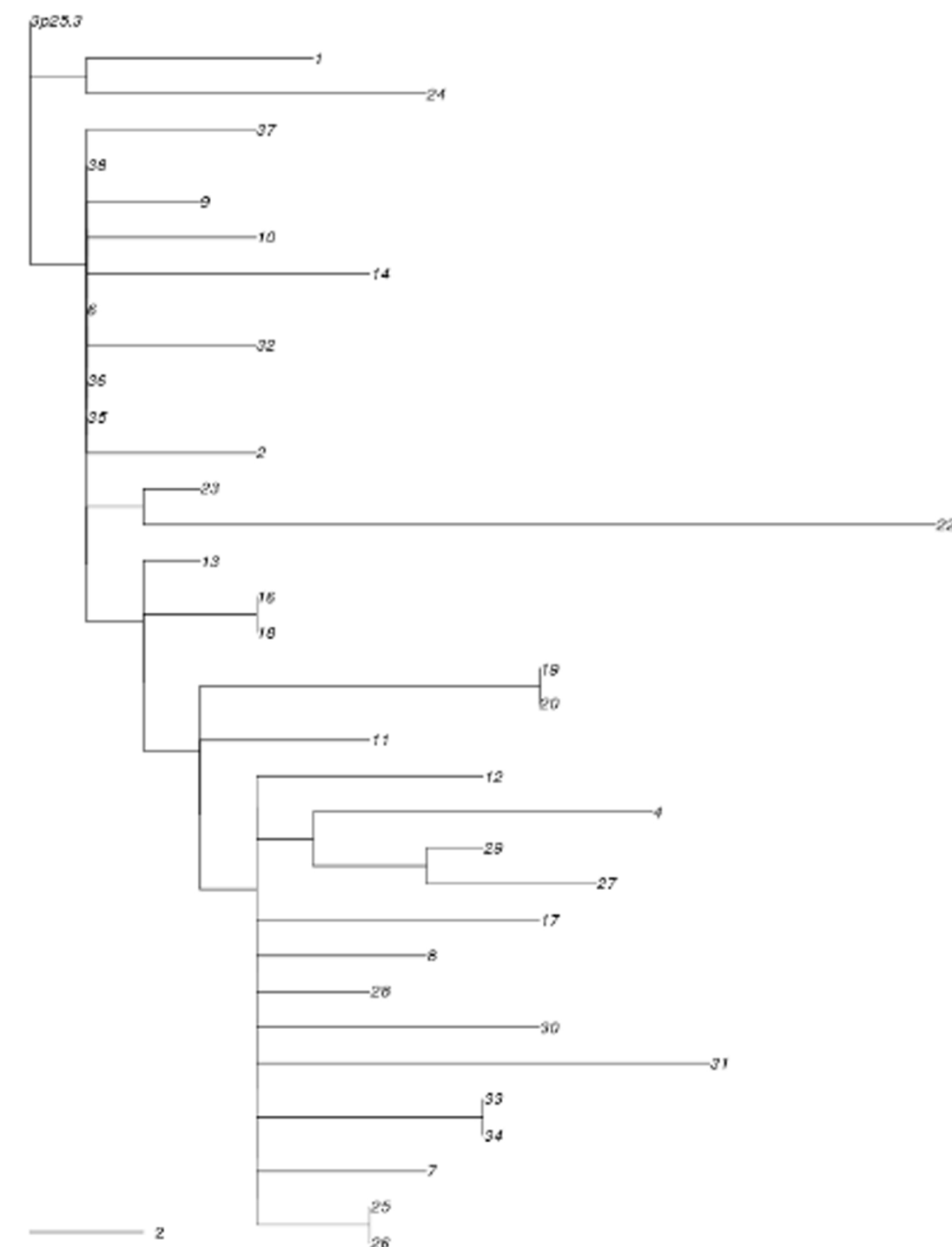

Supplement: Supplementary file 4 — Figure S2. Highlight plot of HERV-K HML-2 3p25.3 sequences. The highlight plot displays the differences in nucleotide sequence of the provirus 3p25.3 amplified by PCR and sequenced using the PacBio platform. Sequences are compared to provirus 3p25.3 (Acc No. JN675020.1), the master sequence. The plot indicates nucleotide substitutions T (red ticks), A (green ticks), C (blue ticks), and G (yellow ticks). A neighbor joining phylogenetic tree is indicated at the right side. The highlight plot was generated using Highlighter from Los Alamos HIV Sequence Database https://www.hiv.lanl.gov/content/sequence/HIGHLIGHT/highlighter_top.html. This indicates that variation in fixed endogenous viral sequence occurs mostly by spontaneous mutations over time rather than homologous recombination which is so characteristic of the enormous sequence variation seen in K111 (PDF 3482 kb) [file 12920_2019_505_MOESM4_ESM.pdf]
